# The Evolution of Grain Microstructure in Friction Stir Welding of Dissimilar Al/Mg Alloys with Ultrasonic Assistance

**DOI:** 10.3390/ma17133073

**Published:** 2024-06-22

**Authors:** Junjie Zhao, Bo Zhao, Chuansong Wu, Sachin Kumar

**Affiliations:** 1 School of Materials Science and Engineering, Shandong Jianzhu University, Jinan 250101, China; zhaojj23@sdjzu.edu.cn (J.Z.); zhaobo19@sdjzu.edu.cn (B.Z.); 2 MOE Key Lab for Liquid-Solid Structure Evolution and Materials Processing, Institute of Materials Joining, Shandong University, Jinan 250061, China; 3 Department of Mechanical Engineering, Indian Institute of Science (IISc), Bengaluru 560012, India; sachinkumar2@iisc.ac.in

**Keywords:** friction stir welding, ultrasonic vibration, grain evolution, aluminum alloys, magnesium alloys

## Abstract

The process of grain refinement during welding significantly influences both the final microstructure and performance of the weld joint. In the present work, merits of acoustic addition in the conventional Frictions Stir Welding (FSW) process were evaluated for joining dissimilar Al/Mg alloys. To capture the near “in situ” structure around the exit hole, an “emergency stop” followed by rapid cooling using liquid nitrogen was employed. Electron Backscatter Diffraction analysis was utilized to characterize and examine the evolution of grain microstructure within the aluminum matrix as the material flowed around the exit hole. The findings reveal that two mechanisms, continuous dynamic recrystallization (CDRX) and geometric dynamic recrystallization (GDRX), jointly or alternatively influence the grain evolution process. In conventional FSW, CDRX initially governs grain evolution, transitioning to GDRX as material deformation strain and temperature increase. Subsequently, as material deposition commences, CDRX reasserts dominance. Conversely, in acoustic addition, ultrasonic vibration accelerates GDRX, promoting its predominance by enhancing material flow and dislocation movements. Even during the material deposition, GDRX remains the dominant mechanism.

## 1. Introduction

The pursuit of lightweight structures is a prominent focus in the advancement of the modern vehicle industry. Consequently, there is a growing utilization of lightweight metals like aluminum alloys and magnesium alloys. The hybrid components/structures made of Mg and Al alloys can combine the properties of both metals together and make full use of their advantages simultaneously [1]. Utilizing these materials in manufacturing processes places greater emphasis on welding technologies. Friction stir welding (FSW) stands out due to its distinctive benefits in joining similar or dissimilar lightweight metals, leveraging a lower heat input and significant plastic deformation [1,2,3]. As a solid-state joining technique, FSW not only circumvents defects commonly encountered in fusion welding such as hot cracks, pores, and coarse grains but also notably mitigates the formation of extensive intermetallic compounds [4,5]. Concurrently, the intense plastic deformation experienced by materials during FSW effectively refines the original grain structure, enhancing structural uniformity [5,6,7]. The performance of welded joints is intricately linked to the grain structure and microstructure within the weld. Consequently, investigating the evolution of grain structure during the welding process emerges as a crucial approach to optimizing welding procedures, regulating weld microstructure, and enhancing joint performance.

In the FSW process, severe plastic deformation and high temperature can drive the dynamic recovery and recrystallization of the base material grains, thereby obtaining refined grains [8]. Various welding thermal-force processes and material characteristics result in distinct grain recrystallization mechanisms. In the FSW of similar Al or Mg alloys, mechanisms such as continuous dynamic recrystallization (CDRX), discontinuous dynamic recrystallization (DDRX), or geometric dynamic recrystallization (GDRX) may significantly contribute to the refinement of the original grain structure. Furthermore, these mechanisms might operate alternately or concurrently [7,8,9,10,11,12].

For the FSW process of dissimilar Al/Mg alloys, although the generation of intermetallic compounds has an important impact on the joint strength, the change in grain structure also has a critical impact on joint performance, such as corrosion resistance [13]. Therefore, it is necessary to study the grain refinement process in FSW of dissimilar Al/Mg alloys. In addition, although a lot of research has been carried out on grain refinement in the process of FSW of similar materials, there is still a lack of relevant research on dissimilar materials’ FSW, which has obvious differences in the welding thermal process.

In order to further improve weld structure and the joint property, ultrasonic vibration (UV) was used in the FSW process, that is, a new ultrasonic vibration-assisted FSW process has been developed. Moreover, various UV-assisted FSW processes have been extensively studied [7,14,15,16,17,18]. A large number of experimental results indicate that the application of UV can effectively improve the microstructure of a weld and the strength of a joint. In the process of metal plastic deformation, the excitation of UV can promote dislocation motion and reduce the activation energy of atomic motion [16]. Therefore, it will definitely have an impact on the evolution process of grain structure. In the previous work of the author, it was also found that in FSW of Al alloy and Mg alloy, the application of UV can promote the recrystallization behavior of grains in the weld nugget zone (WNZ) [18]. However, research about the grain evolution in Al/Mg FSW is based on welds that have finished the welding process. There is a lack of research on how UV affects grain refinement or evolution during welding process.

Given the lack of research on grain microstructure evolution as materials flow around the pin (exit hole) during Al/Mg FSW, as well as the role and influence of ultrasonic vibration in this process, this topic still needs to be explored. This study involved conducting experiments on dissimilar Al/Mg alloys using both conventional and UV-enhanced FSW (UVeFSW). To capture the “near in situ” structure around the exit hole, a combination of “emergency stop” and liquid nitrogen cooling technology was employed during welding. The grain evolution process and its mechanism during the welding process were analyzed through Electron Backscatter Diffraction (EBSD) characterization of grains around the exit hole, with particular attention given to the influence of ultrasonic vibration. It is worth mentioning that in the author’s previous work [19], Mg alloy materials were found to be confined to a narrow region when circulating around the pin. This posed a challenge for gathering grain microstructure information. Consequently, only the grain microstructure on the Al alloy side was examined in this study.

## 2. Experimental Details

AA 6061-T6 and AZ31B-H24 Mg alloy plates of 3 mm thickness were used for friction stir butt welding experimentation, and these alloy materials are commonly and widely used in the automobile industry. The nominal chemical composition (wt. %) of AA 6061 and AZ31B are Si (0.51)-Fe, (0.20)-Cu, (0.30)-Mn, (0.009)-Mg (1.09)-Cr, (0.13)-Zn, (0.05)-Al (Bal.), and Si (0.016)-Fe, (0.001)-Cu, (0.003)-Mn, (0.44)-Mg, (Bal.)-Zn, (1.10)-Al (3.05), respectively. And, the ultimate tensile strength (UTS) of AA 6061 and AZ31B Mg are 294 and 260 MPa, respectively. Based on previous published reports [20], the optimum parameters were chosen as follows: welding speed 50 mm/min, tool rotation speed 800 rpm, AZ31B Mg alloy plate at advancing side (AS), 6061 Al alloy plate at retreating side (RS), and a pin offset 0.3 mm to the Mg side. The welding length was 70 mm. Moreover, the optimized joint tensile strengths could reach 175 MPa (FSW) and 196 MPa (UVeFSW) [20].

**Figure 1:**
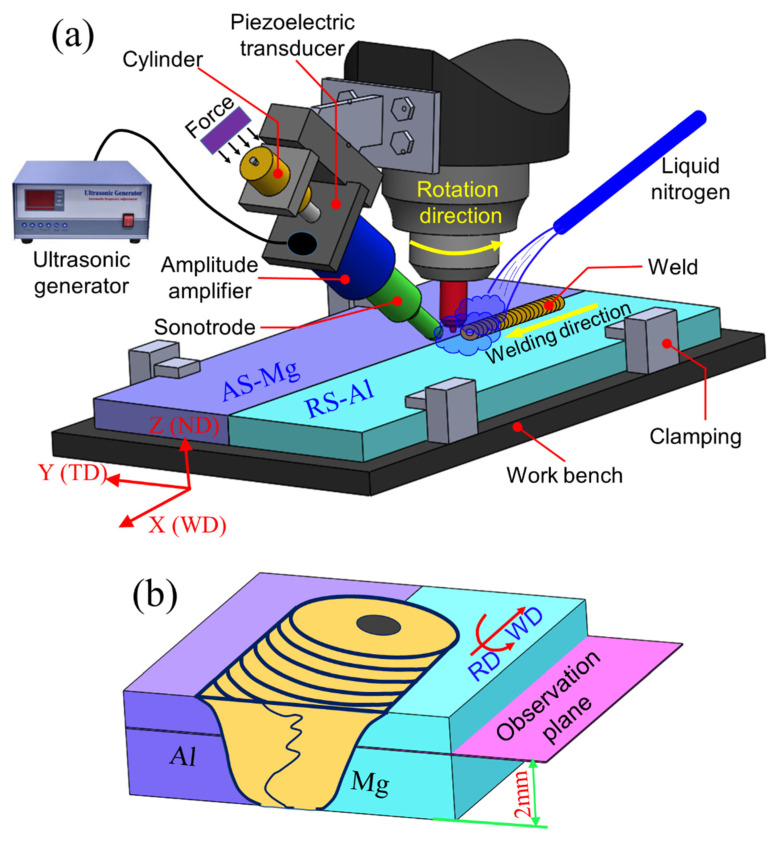
Schematic diagrams of (**a**) ultrasonic vibration enhanced FSW system; (**b**) the sampling position and the horizontal observation plane.

As shown in Figure 1a, the acoustic effects were added in front of the tool at a predefined angle ~ 40° to the workpiece. The distance between horn tip and tool pin was 20 mm. The ultrasonic system works at about 220 W power and 20 kHz frequency.

A tool with a concave geometry shoulder was made of H13 tool steel. It had a shoulder diameter of 12 mm. The tool pin had a frustum shape with right-handed threads. The pin length was 2.8 mm, and its tip and root diameters were 3.2 mm, 4.2 mm, respectively. During welding, the shoulder plunge depth was kept at 0.15 mm, and the tool was inclined by 2.5° opposite to the welding direction.

To gather “in situ” details of the microstructure around the exit hole, the “emergency stop” with an additional liquid nitrogen cooling system was applied to the welding process [8,21]. The weld samples were sectioned around the exit hole, and the horizontal plane 2 mm above the bottom surface of the base plate was selected as the observation surface.

Firstly, the samples were grounded by emery papers and polished by 0.5 μm diamond slurry. Then, an ion beam slope cutter (Leica EM tic 3 ×) was used to remove the stress layer on the sample surface. Finally, a field emission scanning election microscope (FE-SEM) (Zeiss Gemini 500) was used to collect grain microstructure information on the AA 6061 side around the exit hole.

## 3. Experimental Results

### 3.1. Grain Microstructure around the Exit Hole

**Figure 2:**
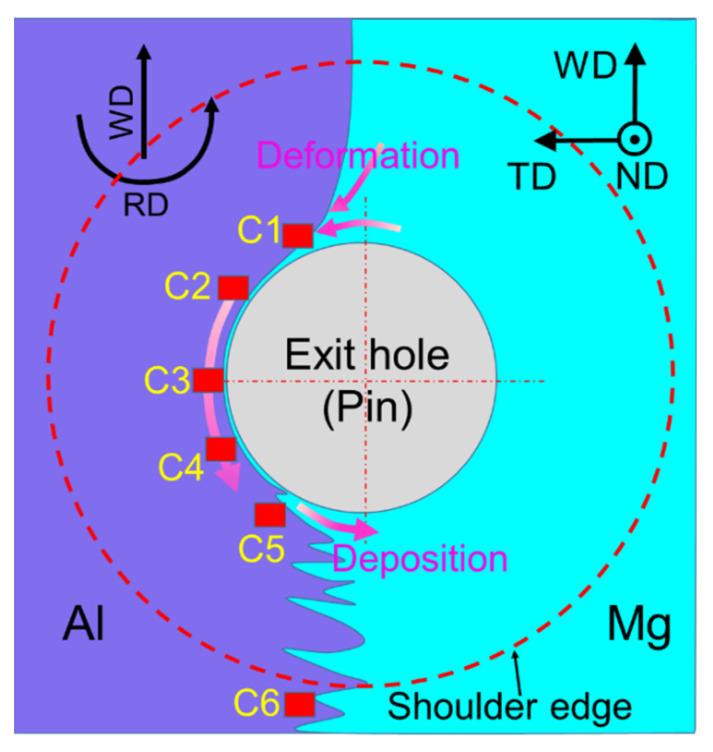
Schematic diagram of EBSD sampling locations.

A schematic of the weld macrostructure around the exit hole is given in Figure 2. The macro- and micro-morphology of the weld region around the exit hole have been given in the author’s previous work [19]. During the welding process, both the Al and Mg metal interfaces associated with the rotating tool gradually deformed under the shearing action of the tool, resulting in a rise in localized temperature. Under plastic deformation, Mg at the AS flows towards the RS as strips. Thereafter, Al and Mg flow around the pin under shearing and tool extrusion. Finally, at the trailing side (TS) of the pin, the flow ability of the materials weakens, and the intermixed materials begin to solidify as the driving ability of the tool decreases. Consequently, mechanical interlocking between the Al and Mg interfaces is gradually developed. Under the influence of UV, the overall appearance of the weld near the exit hole remains relatively unchanged, yet it enhances the material flow around the pin and encourages greater participation of the materials deformation. As the degree of material deformation in UVeFSW is significantly higher compared to conventional FSW [19], it is therefore taken into account for further exploration. In this work, we study the refining process of grain structure in the welding process in order to better understand the effect of UV on grain evolution.

Around the exit hole, along the flow path of the material from the LS to TS, five positions, C1–C5, were marked to characterize the grain microstructure evolution on the Al side. One more position, C6, was also taken into consideration to study the grain microstructure for the region away from the affected tool shoulder region (Figure 2). These C1–C6 locations are shown with red rectangles in Figure 2.

**Figure 3:**
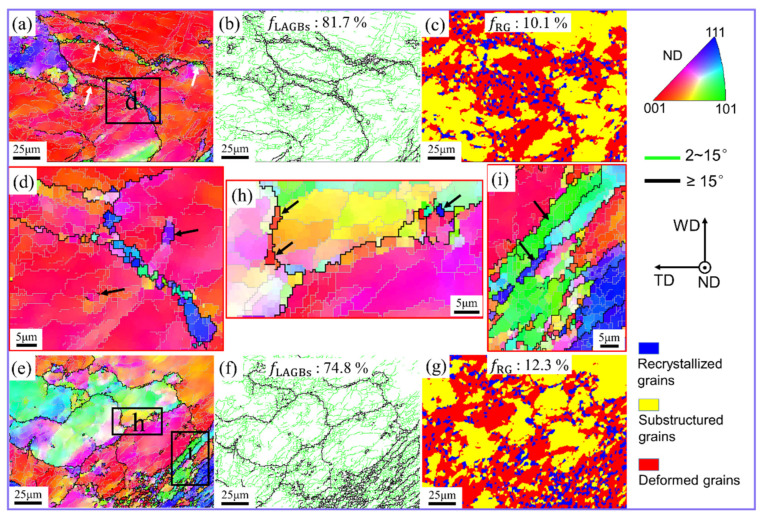
IPF maps (**a**,**d**,**e**,**h**,**i**), GB maps (**b**,**f**), and grain morphology maps (**c**,**g**) at position C1. (**d**,**h**,**i**) Locally enlarged images in (**a**,**e**), respectively. (**a**–**d**) FSW; (**e**–**i**) UVeFSW.

At the initial stage of material deformation given by position C1, the grain microstructure details are given in Figure 3. In the IPF and grain boundary (GB) images, high-angle grain boundaries (HAGBs) and low-angle grain boundaries (LAGBs) are represented by black and silver-gray lines, respectively. Moreover, the critical grain misorientation angle of HAGBs and LAGBs is 15°. For conventional FSW, C1 contains large grains and fine recrystallized grains (Figure 3a). These fine grains are mainly distributed at the boundaries of large grains, forming a typical necklace structure. At the early stage of material deformation, the deformation strain and temperature of the material increase rapidly under the pin shearing. At this stage, multiples dislocations were produced and roamed around the grains. As the material continued to experience high temperature and deformation, these dislocations moved and rearranged to form LAGBs in the large grains, as shown in Figure 3b. At the same time, the original GBs blocked the dislocations motion, causing them to pile-up on each other. Consequently, more LAGBs formed near the original GBs, promoting the conversion of LAGBs to HAGBs by absorbing accumulated dislocations. Thus, the recrystallized grains first formed near the GBs (Figure 3a,d). The grain morphology image also confirms that the recrystallized grains are mainly distributed near GBs (Figure 3c). In addition, there were some HAGBs generated by the gradual accumulation of disorientated angles in the near grain boundaries, as shown by the black arrow in Figure 3d. The recrystallization process from LAGBs to HAGBs is a typical feature of continuous dynamic recrystallization (CDRX) [9].

The UVeFSW sample (region C1) is also composed of large grains and fine recrystallized grains. And, a large amount of LAGBs are also visible in the large grains, as shown in Figure 3e,f. The primary source of formation of these LAGBs is continuous pile-ups of accumulated dislocations. The only difference is that the recrystallized grains are not principally distributed at the boundaries of larger grains but remained at the bottom right-hand corner (Figure 3e), that is, near the pin. From the grain morphology, it is noted that the grains surrounded in the pin vicinity were more recrystallized compared to the farther ones (Figure 3g). The driving force of material deformation mainly came from the shearing action of the pin. Hence, the strain and temperature of material deformation had gradient differences and varied from maximum to minimum as they moved away from the pin. In the sub zone “h” (far away from the pin affected region, shown by black-colored arrows), although grains were recrystallized, their frequency is comparatively lower (Figure 3e,h). The formation of these recrystallized grains could be explained on the basis of the CDRX mechanism.

In the sub-region closer to the pin, the matrix grains appeared to be elongated, which may have been due to the applied strain (shown by the black arrow in Figure 3i). This shows that the geometric effect of strain has an important impact on the grain refinement process. Comparing the grains at position C1 in FSW and UVeFSW, it is found that although CDRX plays an important role in grain refinement in both cases, the geometric effect of strain in UVeFSW also plays a crucial role. This is because UV can promote higher material deformation in the WNZ, as seen in previous reports [22,23,24].

**Figure 4:**
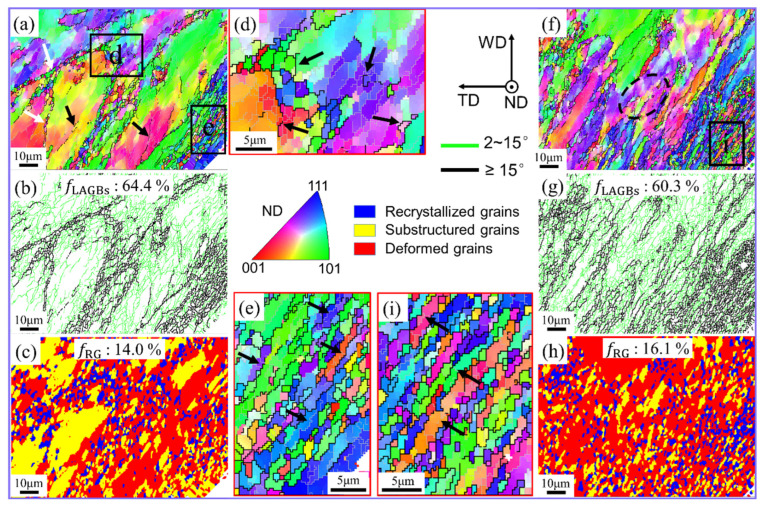
IPF maps (**a**,**d**,**f**,**e**,**h**,**i**), GB maps (**b**,**g**), and grain morphology maps (**c**,**h**) at position C2. (**d**,**h**,**i**) Local enlarged images in (**a**,**f**), respectively. (**a**–**e**) FSW; (**f**–**i**) UVeFSW.

Compared to C1, region C2 experiences a higher temperature, strain, and strain rate, resulting from higher material deformation [10,25]. In conventional FSW, with increased material deformation strain, the base metal grains were elongated and further refined under the geometric effect of strain. Although the outline of the original grain can still be seen, a large number of fine recrystallized grains have been produced on or near GBs, as shown in Figure 4a,b. The higher-magnification image of sub-region d (Figure 4d) shows that the boundary characteristics of the original grains had disappeared and a large number of HAGBs were formed by gradual intensification of the misorientation angle, as shown by the black arrow in Figure 4d. This depicts that CDRX is still an important mechanism for grain refinement in region C2. In the position adjacent to the pin, that is, at the bottom right-hand corner of Figure 4a, the base metal grains are converted into fine fibrous grains due to the influence of severe plastic deformation of the material (Figure 4e). These grains apparently have a comparatively larger aspect ratio, and they are further divided into several sub-grains of similar orientation by LAGBs. A possible cause of this may be the high strain experienced by the affected grains (shown by the black arrow in Figure 4e). Thus, geometric dynamic recrystallization (GDRX) significantly influences grain refinement in this context [8,25,26].

In the UV-treated region in C2, the grains were further elongated into strips, and the boundary contour features of the original grains were essentially no longer visible, as depicted in Figure 4f. This is because of enhanced material plasticization and subsequent deformation under the high strain rate. As the distance from the pin is increased, (from the bottom right-hand corner to the top left-hand corner (Figure 4f–h)), the degree of grain fragmentation and recrystallization decreased gradually due to the reduction in applied strain/strain rate. However, in conventional FSW, we could not notice any obvious gradient change in the distribution of recrystallized grains (Figure 4c). This demonstrates the significant impact of geometric strain on grain refinement in UVeFSW. Sub-region i, located near the pin, was specifically chosen for closer examination, as depicted in Figure 4i. The grain morphology at this location resembled that of conventional FSW, characterized by fine fibrous grains. Moreover, these grains were segmented into multiple sub-grains with similar orientations by LAGBs, as indicated by the black arrow in Figure 4i. This indicates the significant impact of GDRX on grain refinement at this stage. However, unlike in conventional FSW, where GDRX primarily affects grains near the pin, in UVeFSW sample, GDRX emerges as the predominant recrystallization mechanism across the entire observation area. Conversely, in the FSW sample, GDRX primarily influences grains close to the pin, while CDRX governs grain evolution further away from the pin. CDRX also plays a role at position C2 in the UVeFSW sample. As illustrated by the black dotted circle in Figure 4f, the rearrangement of dislocations and the gradual accumulation of grain misorientation angles lead to the generation of some HAGBs.

**Figure 5:**
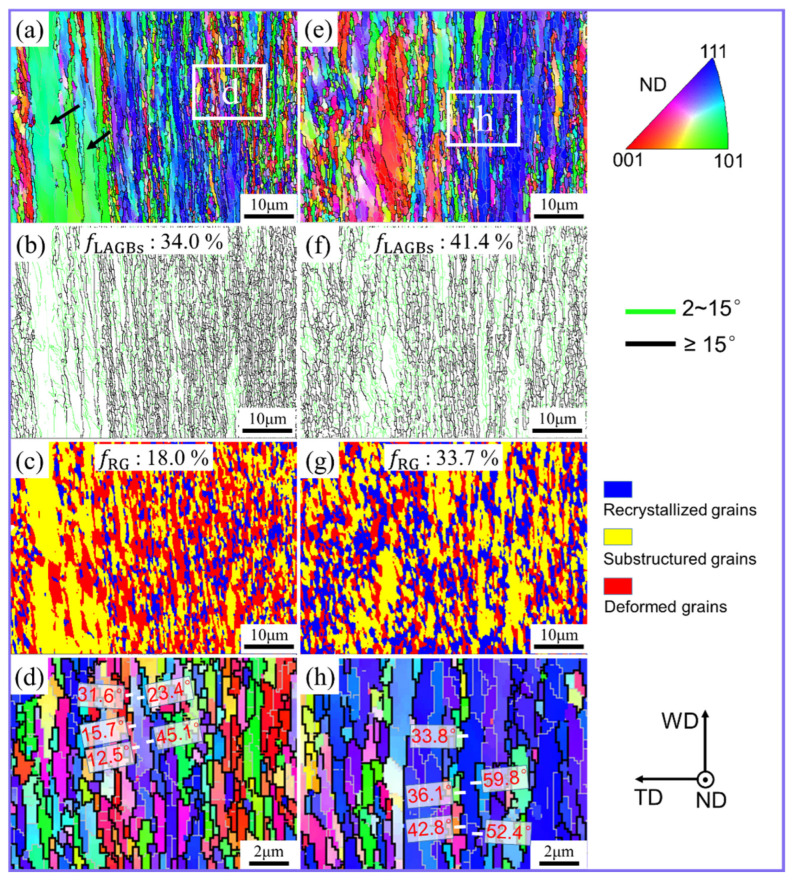
IPF maps (**a**,**d**,**e**,**h**), GB maps (**b**,**f**), and grain morphology maps (**c**,**g**) at position C3. (**d**,**h**) Local enlarged images in (**a**,**e**), respectively. (**a**–**d**) FSW; (**e**–**h**) UVeFSW.

The material at region C3 experiences higher plasticization and more grain refinement, owing to the further rise in localized temperature and strain dependent on the tool rotation direction. As a result, the material flow velocity reaches its maximum value [25,27,28]. In this severe shear deformation region, (Figure 5), the grains in conventional FSW and UVeFSW were both elongated into fibers, while the majority of them were subdivided into fine grains. This shows that the geometric effect of strain dominated grain refinement at this time. Moreover, it can also be seen from Figure 5b,f that position C3 had a high proportion of HAGBs in both the conventional FSW and UVeFSW samples. However, the degree of recrystallization here was relatively low, as shown in Figure 5c,g. In conventional FSW, the area proportion of recrystallized grains was only 18.0%. After the application of UV, the recrystallization degree increased to 41.4%.

In order to investigate the grain microstructure in more detail, sub-zones “d” and “h” were marked for magnification observation. For conventional FSW, the boundary misorientation angle between grains on both sides was calibrated, as marked by white lines in Figure 5d. It was found that the misorientation angle of some HAGBs was about 15.7~23.4°. These boundaries should be formed by the rearrangement of dislocations and the continuous accumulation of misorientation angles during the plastic deformation of materials, causing CDRX [9,25]. The misorientation angles of other HAGBs were about 31.6~45.1°. These HAGBs are often referred to as geometrically necessary boundaries, and they are generally subdivided from the original grains, or related to shear slip [10,29,30,31].

For the sub-zone “h” on the UVeFSW sample, the misorientation angles of a few HAGBs were also calibrated, as marked by white lines in Figure 5h. The misorientation angle of these HAGBs was about 33.8~59.8°, which is higher compared to conventional FSW. This may be due to difference in the grain refinement processes. In conventional FSW, the CDRX mechanism has always played an important role in the grain refinement process before materials flowed to position C3, so some HAGBs generated by the gradual accumulation of misorientation angles can be found. For UVeFSW, GDRX dominated the grain refinement process at position C2, and most HAGBs of the new grain were inherited from the original grain boundaries. Therefore, they generally have a high misorientation angle value.

In addition, it is also worth noting that in conventional FSW, the grain refinement degree and recrystallization grain distribution in position C3 show obvious gradient changes with the increase in the distance from the pin, as shown in Figure 5a–c. However, this phenomenon does not exist in UVeFSW, as shown in Figure 5e–g. This proves that the application of UV can promote more materials to participate in deformation, which is consistent with the previous results observed in metallographic and SEM investigations [21].

**Figure 6:**
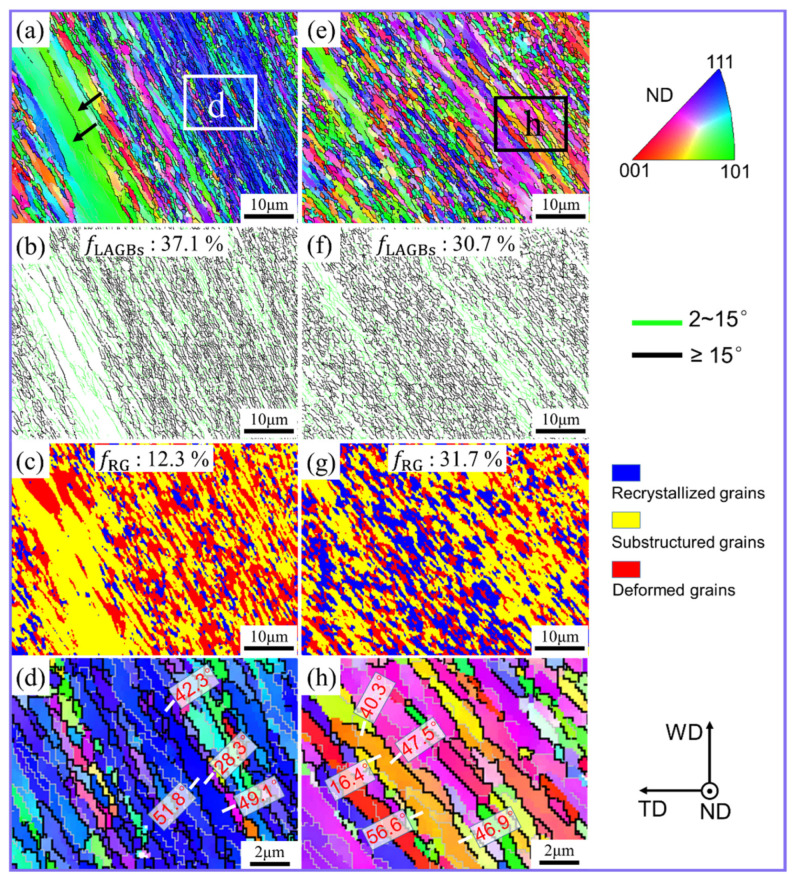
IPF maps (**a**,**d**,**e**,**h**), GB maps (**b**,**f**), and grain morphology maps (**c**,**g**) at position C4. (**d**,**h**) Local enlarged images in (**a**,**e**), respectively. (**a**–**d**) FSW; (**e**–**h**) UVeFSW.

The grain microstructure at region C4 for both UV and non-UV treated samples is given in Figure 6. Interestingly, the grain microstructure at C4 is pretty identical to that of region C3, which is composed of several fine fibrous grains. HAGBs account for a high proportion in both conventional FSW and UVeFSW, as shown in Figure 6a,b,e,f. In conventional FSW, a few large lath grains of identical structure are evident (Figure 6a), which remain absent in UVeFSW (Figure 6e). This shows that the addition of UV contributes to the uniformity of grain microstructure evolution at position C4.

From positions C3 to C4, the material is still at an elevated temperature [25,27]. At this stage, the dynamic recovery and recrystallization of the affected grains should be greater compared to other regions. Meanwhile, the degree of grain recrystallization at position C4 was slightly lower than that at position C3 in conventional FSW, and a similar phenomenon occurred in UVeFSW, as shown in Figure 5c,g and Figure 6c,g. This phenomenon is bound to the severe plastic deformation of materials. Previous reports have suggested that when the materials flow through the position perpendicular to the welding direction (corresponding to position C3), the deformation temperature and strain rate will be at their peak values [25,27,28]. However, due to a 2.5° tool tilt angle, the material flow at position C4 can still maintain a high flow velocity under the shearing and forging action of the shoulder and the pin. Therefore, continuous severe plastic deformation causes the continuous generation of new sub-grains in the Al matrix, thus affecting the degree of grain recrystallization. At position C4, the excitation of UV can still significantly promote the recrystallization behavior of grains.

For in-depth investigation, the sub-zones “d” and “h” at position C4 were selected for conventional FSW and UVeFSW samples, respectively (Figure 6a,e). In conventional FSW, different from position C3, the misorientation angles of the selected HAGBs belong to one grain there, and all have a large value, as marked by white lines in Figure 6d. Moreover, the grains have a higher aspect ratio than those at position C3. It can also be seen that the grain boundaries were mostly serrated, and some LAGBs divided these grains into sub-grains with approximate orientation. This shows that grain refinement is almost not affected by CDRX, while GDRX plays a leading role [8,25,32]. The grain microstructure in UVeFSW has a similar morphology to that in FSW, that is, GDRX dominates the grain evolution at position C4.

**Figure 7:**
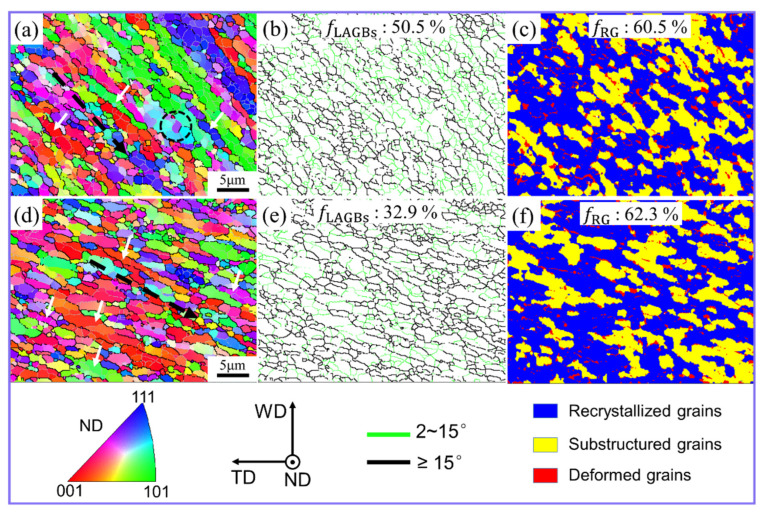
IPF maps (**a**,**d**), GB maps (**b**,**e**) and grain morphology maps (**c**,**f**) at position C5. (**a**–**c**) FSW; (**d**–**f**) UVeFSW.

As the material reached position C5, its flow velocity slowed down, initiating deposition [10,19,25,27,28]. Nonetheless, the grains retained evident deformation orientation, as illustrated by the black dotted arrow in Figure 7a,d. Additionally, LAGBs divided most grains into multiple sub-grains with comparable orientations, as depicted by the white arrows in Figure 7a,d. This shows that the grain evolution here was still affected by GDRX. It is worth noting that compared with positions C3 and C4 (Figure 4b and Figure 5b), the grains at position C5 have massive LAGBs (about 50.5%) in the conventional FSW (Figure 7b). Moreover, the sub-grains formed by these LAGBs have a small aspect ratio. This also shows that the strain rate of material plastic deformation decreased at position C5.

At the same time, the LAGBs formed some sub-grains through the rearrangement of dislocations and the gradual accumulation of misorientation, as shown by the black dotted circle in Figure 7a. This shows that with reduction in the material deformation strain rate, CDRX is poised to exert a significant influence on the grain evolution process once more. Furthermore, it becomes apparent that during the FSW process, grain refinement is not solely influenced by a singular recrystallization mechanism but rather by the synergistic or alternating effects of multiple mechanisms. Ji et al. [6] also pointed out that GDRX, CDRX, and discontinuous dynamic recrystallization (DDRX) simultaneously or alternately affect the grain recrystallization process during FSW of Al-Mg-Si alloy. Moreover, because the formation of grain boundaries requires the continuous accumulation of dislocations during the CDRX process, a large number of LAGBs are generated, which is why position C5 in conventional FSW shows a large number of LAGBs. However, position C5 in UVeFSW contains fewer LAGBs, as shown in Figure 7e. This aligns with the observation that UV tends to facilitate dislocation motion and rearrangement, thereby promoting the absorption of dislocations through LAGBs and their transformation into HAGBs [7,16]. Furthermore, in both the conventional FSW and UVeFSW samples, there is a notable prevalence of recrystallized grains at position C5, as illustrated in Figure 7c,f. This can be attributed to the higher accumulated energy within the grains during the flow process. Additionally, although the deformation strain rate of the materials decreased, the elevated temperature remained conducive to dynamic recovery and dynamic recrystallization.

**Figure 8:**
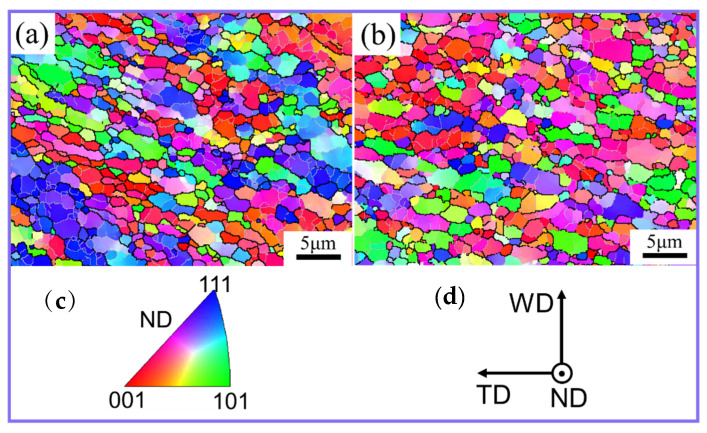
IPF maps (**a**,**c**) and grain morphology maps (**b**,**d**) at position C6. (**a**) FSW; (**b**) UVeFSW.

When the material flowed outside of the contour range of the shoulder, the material deformation stopped and the temperature decreased rapidly. The grain microstructure at position C6 is shown in Figure 8. Whether conventional FSW or UVeFSW, position C6 was almost entirely composed of fine equiaxed grains. And the average grain size was ~1.4 μm and ~1.1 μm for FSW and UVeFSW, respectively. The application of UV can slightly refine grain size. This conclusion is consistent with the author’s previous research on the effect of UV on grain evolution in the WNZ [18].

It is worth noting that the materials were still deforming when deposition began due to the forging and shear action of the shoulder [19], which made the Al/Mg materials interpenetrate and intermix with each other, forming a tortuous and complex interface structure. However, the degree of plastic deformation in the process was not intense, and the evolution of grain structure was closer to an annealing process. Therefore, LAGBs were gradually transformed into HAGBs, the recrystallization degree of grains was further improved, and the grain size was also slightly increased [8].

### 3.2. Evolution of Grain Microstructure Information

**Figure 9:**
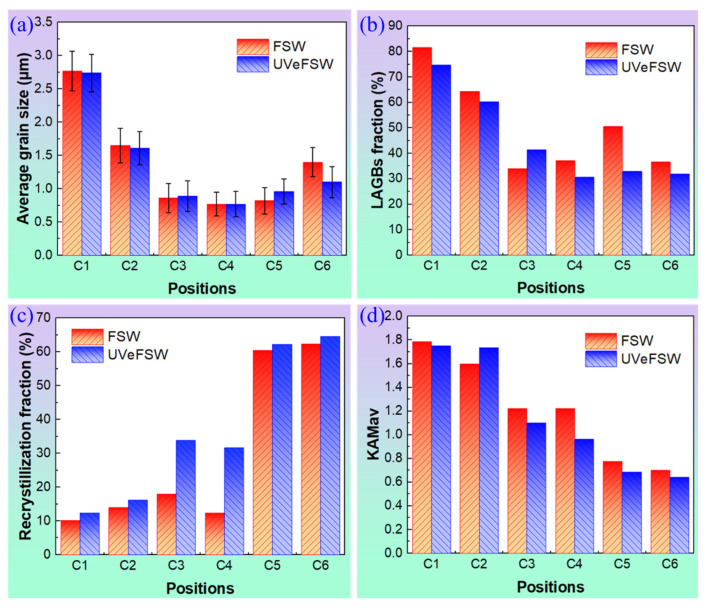
Grain microstructure evolution information at positions C1–C6 on FSW and UVeFSW samples. (**a**) Recrystallized grain fraction. (**b**) LAGB fraction. (**c**) Average grain size. (**d**) Average KAM value.

In order to better analyze the evolution process of grains around the exit hole and the effect of UV, the grain microstructure information at positions C1–C6 in conventional FSW and UVeFSW was collected, as shown in Figure 9. From positions C1 to C3, the temperature and the deformation strain/strain rate of materials increased gradually. The original base metal grains underwent gradual refinement into finer grains through dynamic recovery and dynamic recrystallization. Consequently, the average grain size exhibited a gradual decrease from positions C1 to C3, as depicted in Figure 9a. Simultaneously, as dislocations accumulated and rearranged, and dynamic recrystallization took place, the extent of grain recrystallization continued to rise. This was evidenced by the gradual decrease in the number fraction of LAGBs and the average KAM value, as illustrated in Figure 9b–d.

From positions C3 to C4, the LAGB fraction, average grain size, and average KAM value changed slightly, and the recrystallization degree of grains even decreased. This phenomenon arises because severe plastic deformation continuously generates new dislocations and sub-grains despite the material being at a high temperature. This dynamic process “hinders” the progression of dynamic recovery and dynamic recrystallization. It can also be interpreted that dynamic recovery and dynamic recrystallization approach an approximate “equilibrium” state due to the work hardening of the material. This is consistent with the previous conclusion obtained from the analysis of grain morphology (Figure 5 and Figure 6).

From positions C4 to C5, the deformation strain rate and the flow velocity of the materials around the pin decreased, and the dynamic recovery and dynamic recrystallization process played a leading role in this case. The degree of grain recrystallization was significantly improved, and the average KAM value also decreased.

From positions C5 to C6, the grain size increases and the LAGB fraction decreases due to the deposited material still existing at a high temperature [25]. However, these materials were still within the contour range of the shoulder, and the forging effect of the shoulder could cause slight plastic deformation of these materials [19]. Therefore, the degree of variation in the recrystallization fraction and the average KAM value are relatively small.

Although the evolution of grain microstructure was roughly the same as that in conventional FSW, there were two obvious differences with the application of UV. First, at positions C3 and C4, the recrystallization degree of grains was quite different between conventional FSW and UVeFSW, as shown in Figure 9b. The area fraction of recrystallized grains in UVeFSW was significantly higher compared to conventional FSW. The process of grain recrystallization is closely linked to both the deformation strain rate and the temperature of the material [33]. Previous experimental findings by the author have demonstrated that the activation of ultrasonic vibration has minimal impact on the temperature of the WNZ [34]. Therefore, the role of temperature in this variation can be discounted. However, numerous experimental results have established that the application of ultrasonic vibration enhances material flow within the WNZ [20,24,26]. In UVeFSW, the material experiences greater deformation strain/strain rate as it flows around the pin on the RS, thereby facilitating increased energy storage for dynamic recovery and dynamic recrystallization. Moreover, during the process of metal plastic deformation, UV can promote the motion and rearrangement of dislocations [16], which is conducive to dynamic recovery and dynamic recrystallization. In addition, it was found from the analysis of grain micro-structure above that at position C3 in FSW, CDRX and GDRX jointly affected grain evolution. Meanwhile, GDRX dominated grain refinement in UVeFSW. This difference in the grain evolution mechanisms also has an important effect on the recrystallization of grains.

**Figure 10:**
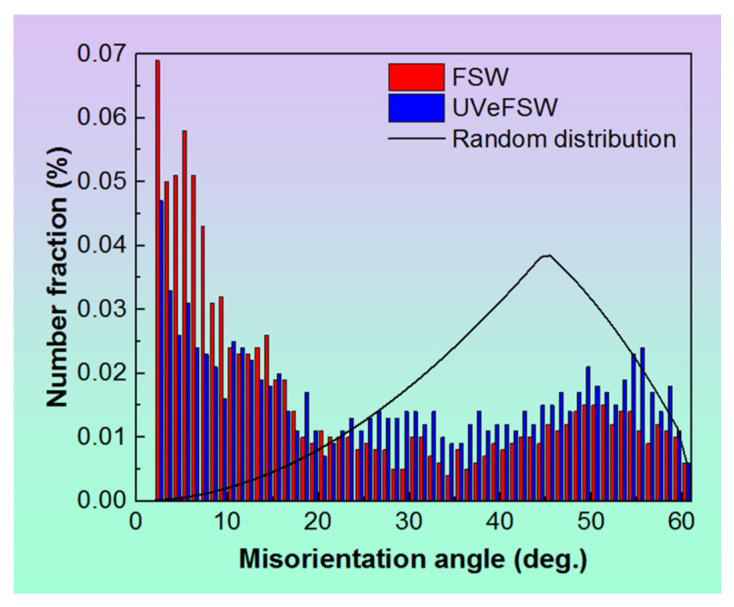
Histogram of grain misorientation angle distribution at position C5 in FSW and UVeFSW.

Another big difference caused by UV was the LAGB fraction at position C5, as shown in Figure 9c. The value in UVeFSW was significantly lower than that in conventional FSW. Typically, the smaller the LAGB fraction, the higher the recrystallization degree of the affected grains. However, the extent of grain recrystallization at position C5 in conventional FSW does not show significant divergence from that in UVeFSW, as evidenced in Figure 9b. Upon analyzing the grain microstructure above, it became apparent that GDRX exerted a significant influence on grain evolution at position C5 in both FSW and UVeFSW. However, CDRX also played a crucial role in FSW. Furthermore, examining the distribution histogram of the grain misorientation angles at position C5, as depicted in Figure 10, reveals that in conventional FSW, the number fraction of boundaries with misorientation angles less than 10° is higher, while it is lower within the misorientation angle range of 25~60°. This indicates that a large number of LAGBs were formed through dislocation rearrangement and accumulation in conventional FSW. The difference in the grain refinement mechanisms is the main reason for this phenomenon. In UVeFSW, the higher deformation strain rate and flow velocity of the material when it flows around the pin were conducive to the occurrence of GDRX. This further proves that UV can promote material flow even at the place where the materials begin to deposit, which is also conducive to the mixing of Al/Mg materials to form a high-quality weld joint. This is consistent with previous macro-microstructure characterization results [10,20].

In addition to these two obvious, above-mentioned differences, the application of UV also affects other grain microstructure information. For example, the average KAM (Kernel Average Misorientation) value of grains at position C2 in UVeFSW was higher than that in conventional FSW, but lower at positions C3–C5, as shown in Figure 9d. KAM images indirectly qualitatively represent the homogenization degree of a material’s plastic deformation and the dislocation density. Indeed, the smaller the KAM value, the lower the dislocation density tends to be [35]. This should be related to the fact that UV can promote material flow and dislocation motion. At position C2, the violent plastic deformation in UVeFSW generated more dislocations. As the materials flowed to positions C3–C5, their temperature increased [25,27], leading to dynamic recovery and dynamic recrystallization playing a predominant role in the process. At this time, the excitation of UV was conducive to dislocation rearrangement and annihilation; thus, the average KAM value of the grains decreased.

## 4. Conclusions

1. During the process of material flow around the pin in Al/Mg FSW and UVeFSW, CDRX and GDRX alternate or jointly affect the grain refinement process of the Al matrix.
2. In conventional FSW, CDRX is dominant during the initial deformation stage. As material flow progresses, the deformation strain/strain rate increases, leading to the onset of GDRX. GDRX becomes the dominant grain refinement mechanism when the material flows through the position perpendicular to the welding direction at the RS (position C3). Upon material deposition, CDRX resumes its role due to the reduction in deformation strain rate.
3. In UVeFSW, CDRX is initially dominant during the initial stage of material deformation. However, as material continues to flow around the pin at the RS, GDRX begins to play a role earlier than in conventional FSW, becoming the dominant mechanism. This dominance persists even at the point where material deposition begins.
4. The influence of ultrasonic vibration on the grain recrystallization process is mainly reflected in the positions C3 and C4. By changing the recrystallization mechanism of the material (CDRX→GDRX), the degree of grain recrystallization is promoted.
5. The difference in evolution is attributed to the fact that UV promotes material flow and dislocation motion.

## Data Availability

Data is contained within the article.
